# Unique Anti-Glioblastoma Activities of Hypericin Are at the Crossroad of Biochemical and Epigenetic Events and Culminate in Tumor Cell Differentiation

**DOI:** 10.1371/journal.pone.0073625

**Published:** 2013-09-16

**Authors:** Naama Dror, Mathilda Mandel, Gad Lavie

**Affiliations:** 1 Department of Cellular and Developmental Biology, Sackler School of Medicine, Tel Aviv University, Tel Aviv, Israel; 2 The Blood Center, Sheba Medical Center, Tel-Hashomer, Israel; University of Portsmouth, School of Pharmacy & Biomedical Sciences, United Kingdom

## Abstract

Failure of conventional therapies to alleviate glioblastoma (GBM) fosters search for novel therapeutic strategies. These include epigenetic modulators as histone deacetylase inhibitors (HDACi), which relax abnormally compact tumor cell chromatin organization, enabling cells to overcome blockage in differentiation. However, in clinical settings, HDACi efficacy is confined to subsets of hematologic malignancies. We reasoned that molecules targeting multiple epigenetic mechanisms may exhibit superior anti-cancer activities. We focused on the redox perylene-quinone Hypericin (HYP) and showed that HYP targets Hsp90 for polyubiquitination, degradation and inactivation. Hsp90 is implicated in mediating inheritable epigenetic modifications transferable to progeny. We therefore examined if HYP can induce epigenetic alterations in GBM cells and show here that HYP indeed, targets multiple mechanisms in human glioblastoma tumor cell lines via unique manners. These elicit major epigenetic signature changes in key developmentally regulated genes. HYP induces neuroglial tumor cell differentiation modulating the cytoarchitecture, neuroglial differentiation antigen expression and causes exit from cell proliferation cycles. Such activities characterize HDACi however HYP is not an HDAC inhibitor. Instead, HYP effectively down-regulates expression of Class-I HDACs, creating marked deficiencies in HDACs cellular contents, leading to histones H3 and H4 hyperacetylation. Expression of EZH2, the Polycomb repressor complex-2 catalytic subunit, which trimethylates histone H3K27 is also suppressed. The resulting histone hyperacetylation and diminished H3K27-trimethylation relax chromatin structure, activating gene transcription including differentiation-promoting genes. DNMT profiles are also modulated increasing global DNA methylation. HYP induces unique epigenetic down-regulations of HDACs, EZH2 and DNMTs, remodeling chromatin structure and culminating in tumor cell differentiation. These modulations generate clinically significant anti-GBM effects obtained in a clinical trial performed in patients with recurrent, progressive disease. Despite this advanced disease stage, patients responded to HYP, displaying stable disease and partial responses; patients on compassionate therapy survived for up to 34 months. Hypericin may constitute a novel anti-glioblastoma therapeutic paradigm.

## Introduction

Therapy of the most aggressive brain cancer, glioblastoma multiforme (GBM), which combines surgery, radio-chemotherapy and post-recurrence immunochemotherapy has failed to relieve patients from disease progression. Overall median survival remains 14.6 months [1]. Treatment objectives thus aim to alter tumor cell properties and explore new molecular paradigms. Some objectives focus on modulating cancer cell gene expression patterns via adjustments of abnormal epigenetic codes, including among others, hypoacetylation of histones H3 and H4, which occur in various malignancies including GBM [2]. They are primarily due to elevated activities of histone deacetylases (HDACs), and cause increased chromatin compaction, diminishing transcription of many genes. Cell differentiation, replication arrest and apoptosis are all inhibited, thereby promoting development of malignancies [3], [4].

Cancer cell transcriptomes are also modified by histone methyltransferases. One such enzyme, Polycomb repressive complex-2 (PRC2) methylates histone H3 to trimethyl-lysine-27 (H3-K27-3me) [5] and is implicated in carcinogenesis. PRC2 catalytic subunit EZH2 is abnormally elevated in several tumors including GBM with highest levels correlating with advanced disease stage and poor prognosis [6]. EZH2 forms physical interactions and functional links with HDACs [7] and with all three DNA methyl transferases (DNMTs) [8], generating aberrant epigenetic machineries that dysregulate gene promoter methylation patterns. Although globally tumor cell DNA is hypomethylated, promoters of tumor suppressor genes become hypermethylated silencing their expression [9], [10]. DNMT1 and DNMT3b expressions are also abnormally elevated in GBM cells [11]–[13].

Since epigenetic aberrations form neoplasia-promoting platforms [14], they can be targets for anticancer therapy aiming to relax compacted cancer cell chromatin, rendering transcription factors accessible to differentiation-related gene promoters [15], [16]. Such goals became achievable through increasing histone acetylation using small molecule histone deacetylase inhibitors (HDACi). HDACi overcome blocks in tumor cell differentiation, reactivate apoptosis and alter angiogenesis [17] however, consistent clinical benefits are confined to subtypes of haematologic malignancies [13]. HDACi effects in solid tumors appear marginal and inconsistent.

One reagent which may potentially be capable of targeting several aberrant epigenetic regulatory functions with better solid tumor therapeutic efficacy is hypericin (HYP) analyzed here. This perihydroxylated perylene quinone displays multiple anticancer activities resulting from a unique ability to induce forced polyubiquitination of Hsp90 in cancer cells [18], [19]. Hsp90 is consequently degraded, destabilizing its plethora of client proteins, many of which are kinases active in signaling pathways. The deficiencies in hsp90 client proteins impair tumor cell replication [18], [19] and have been shown to effectively prevent production of VEGF, the hormone responsible for induction of tumor angiogenesis [20].

Hsp90 has also been reported to link chaperone activities with epigenetic gene regulation in morphological evolutions of Drosophila melanogaster variants and Arabidopsis thaliana [21], interacting with key chromatin remodeling complex components [22]. The modified epigenetic codes are transmitted to progeny through many replication cycles. Here we show that HYP can also exert epigenetic anti-GBM activities and induce post-mitotic GBM tumor cell differentiation, mimicking HDACi effects, although HYP is not an HDACi. The action of HYP is shown to modify aberrant parameters in the tumorigenic epigenetic code, downregulating expression of class I HDACs and EZH2 in GBM tumor cell lines. As a result these actions of HYP may affect functionalities of select DNA methyltransferases since HDACs and EZH2 physically associate and form functional links with DNMTs [8]. We present data which shows that HYP can also reduce abnormal DNMT profiles and may modify aberrant epigenetic platforms and dysregulated oncogenic DNA methylation patterns which promote genomic instability and facilitate GBM oncogenesis [23], [24].

## Materials and Methods

### Cell Culture treatment with Hypericin

U87-MG, T98G and U251-MG human GBM cell lines were obtained from the American Type Culture Collection (ATCC). The cells were cultured in DMEM, 10% FBS, 2 mM L-glutamine, penicillin 100 units/ml, streptomycin 100 µg/ml and grown at 37°C, 5% CO_2_. Hypericin-sodium was synthesized as described in [19] dissolved in 70% ethanol to 2 mg/ml stock solution and further diluted in complete medium. HYP is a potent photodynamic agent and was therefore applied to cultures in darkness (hood lights shut off; ambient light kept ≤0.03 mW/cm^2^ to prevent phototoxicity). Light intensities were quantified using the IL 1350 Radiometer/Photometer from International Light Inc., (Newburyport, MA).

### Quantitative cell viability assays

Cell viability was monitored using the MTT assay, quantified spectrophotometrically at 560 nm subtracting nonspecific absorption at 650 nm. HYP-attributed elevated backgrounds were normalized relative to cell-free blank wells containing medium and each pigment concentration.

### HDAC-mediated deacetylation activity assay

Deacetylation assays were performed using the “Fluor de Lys” substrate (Product BML-KI138, 50 mM in DMSO from ENZO Life Sciences, Ann Arbor MI). This proprietary fluorimetric histone deacetylase lysyl substrate becomes sensitized to the developer and is excited by light at 360 nm following deacetylation of acetylated lysine by HDACs. Cell lysates were prepared and calibrated to 15 µg protein and the assays performed according to manufacturer's instructions. Fluorescence was quantified using Labsystems Fluoroskan Ascent CF following excitation at 355 nm and emission at 460 nm.

### Methylated DNA quantification

DNA was extracted using DNeasy Blood & Tissue kit (QIAGEN GmbH). Total DNA metylation was quantified using the MethylFlash Methylated DNA Quantification kit (colorimetric) (EPIGENTEK) according to manufacturer's instructions.

### Western Blot analyses

Protein lysates were prepared from whole cells treated with HYP (72 hrs), in RIPA buffer containing 40 µg/ml Complete Protease Inhibitor (Roche Diagnostics, Mannheim, Germany) and protein content was calibrated using the BCA protein assay reagent kit (Thermo Fisher Scientific Inc.). Rabbit polyclonal antibodies to GFAP, HDAC1, HDAC2 and HDAC3 were from Abcam, (Cambridge, UK) and used as a 1:500 dilution. Anti-GAPDH (used as a 1:500 dilution) was from Santa Cruz Biotechnology, Rabbit polyclonal anti-H3K27, Mouse monoclonal anti-EZH2, peroxidase-conjugated Affinipure Goat anti Rabbit IgG (H+L) and Peroxidase-conjugated Affinipure Goat anti Mouse IgG (H+L) (both secondary antibodies were from Jackson Immuno Research and were used as a 1:10,000 dilution). Proteins were transferred to nitrocellulose membranes (Schleicher & Schuell, Germany) and Western blots were prepared following standard procedures.

### Quantitive RT-PCR analyses

Cells were treated with HYP for 48 hrs and total RNA extracted using TRIzol reagent, Invitrogen). cDNA was obtained using Verso cDNA kit (Thermo Scientific). PCR was carried out using power SYBR green PCR master mix (Applied Biosystems) with respective primers, detailed in Table S1, in a 7500 Fast Real Time PCR system from Applied Biosystems. Fold increases relative to RPLPO (human large ribosomal protein) endogenous control, were calculated.

### Immunofluorescence and Confocal Microscopy

Cells cultured on glass slides were HYP treated (72 hrs), washed with PBS, fixed with 4% paraformaldehyde, 10 minutes, blocked with 10% donkey serum-1% BSA and incubated with primary antibody (in a dilution of 1:100 in 1% BSA in PBS, 2 hrs). Primary antibodies used from Abcam were: polyclonal rabbit anti-GFAP, anti-acetylated H4 and mouse monoclonal anti 5-methylcytidine. From Millipore: mouse monoclonal anti-tubulin beta-III, anti-NeuN clone A60, rabbit polyclonal anti-acetylated H3. Slides were stained with Alexa Fluor 488-conjugated anti-rabbit secondary antibody (also used in a dilution of 1:1000 for one hour, counterstained with DAPI using Vectashield mount with DAPI and imaged using confocal microscope.

### Colony Formation Assay

Cells seeded 500/well in six-well plates were allowed to attach in medium DMEM-10% FCS overnight. The growth medium was then replaced with a medium containing HYP (0–50 µM) and the cells cultured for 14 days, washed with PBS, fixed with methanol and stained with hemacolor reagents (Merck, KGaA, Darmstadt, Germany). In this assay Hemacolor® Solution 2 (red) is applied onto the cells for one minute, rinsed with PBS and stained with Hemacolor® Solution 3 (blue) for one more minute and rinsed with PBS. Colony formation (efficiency of cell plating to form colonies) was analyzed microscopically by scoring the number of colonies formed, and effects of colony sizes were quantified by extraction of the hemacolor dyes from the cells using 0.5% SDS in PBS incubated for 30 min and the overall number of cells in the colonies quantified by spectrophotometric analysis of the eluted dye in an ELISA reader at 650 nm against a standard curve as described in reference [25].

### siRNA Transfections

Validated siRNA directed against *STAT3* and AllStars Negative siRNA AF 488 were obtained from QIAGEN GmbH (Hilden Germany). Cells were transfected using HiPerfect transfection reagent (QIAGEN GmbH, Hilden Germany) as per supplier's instructions. The cells were exposed to HYP 24 hrs after transfection. At 48 and 72 hrs after transfection cells were washed, harvested for RNA and for protein cell lysates. qRT PCR and Western blot analyses were performed.

### Chromatin immunoprecipitation (ChIP) and quantitative real-time PCR

ChIP assays were performed on U87-MG cells as previously described [26]. Immunoprecipitations were performed using polyclonal antibodies for STAT3 (Millipore) as described [26]. PCR analyses were performed with primer sequences shown in Table S2. Polyclonal rabbit IgG (Millipore) was used as negative control.

## Results

### Down-regulation of GBM tumor cell proliferation by Hypericin

**Figure 1:**
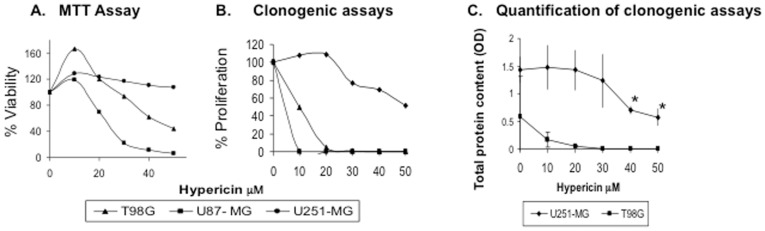
Downregulation of tumor cell proliferation and clonogenicity by HYP in T98G, U87-MG and U251-MG cells. (A) Cell viability analyses following exposure to HYP using the MTT assay. (B) Clonogenicity of these cells following HYP exposure for 14 days. (C) Quantification of cell contents in the clonogenic assays to determine overall number of cells in the clones using the Hemacolor assay.

A most important feature of prospective antitumoral reagents is prevention of tumor cell replication. Thus, effects of HYP on cell proliferation were analyzed in U87-MG, U251-MG and T98G human GBM tumor cell lines. The treatments (for 72 hrs) caused decreases in cell viability monitored by MTT assays (Fig. 1A), and cell proliferation arrest in 14 day clonogenic assays (Fig. 1B). Effects were most effective in U87-MG cells and in T98G cells (Figs. 1A and 1B) however, U251-MG cells were relatively more resistant to anti-proliferative HYP activities. Although colonies which formed after cell exposure to the highest HYP doses of 40 and 50 µM were sparser and smaller compared to untreated controls, these effects were of lesser magnitudes as shown by quantitative cell content analyses, (Fig. 1C). Thus, HYP effectively interfered with cell proliferation in two of three studied GBM tumor cell lines.

### Induction of glioblastoma tumor cell differentiation with Hypericin

**Figure 2:**
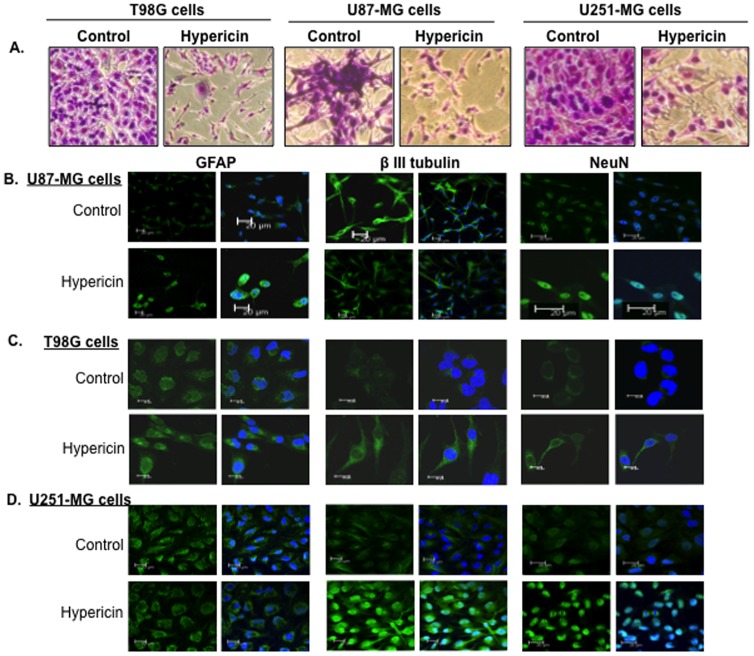
Induction of GBM tumor cell differentiation following exposure of these GBM cell lines to HYP (20 µM). (A) Changes in cell morphology; staining with Hemacolor reagents. (B) Expression of neuro-glial differentiation antigens GFAP (glial), βIII-tubulin (neuronal) and NeuN (early neuronal nuclear) in HYP-treated U87-MG cells, (C) T98G cells, (D) U251-MG cells. Immunocytochemical staining. Right exhibit in each line reflects samples with nuclear counterstaining with DAPI.

The cell proliferation arrest caused by HYP in the GBM cell lines fostered investigation of possible association of this arrest with induction of tumor cell differentiation by HYP. Proof of concept experiments were conducted in the three GBM cell lines treated with HYP (20 µM dose, 72 hrs), fixed, stained with hemacolor reagents for cytoskeletal remodeling analyses and for immunocytochemical analyses to evaluate neuro-glial differentiation antigen expression. Fig. 2A shows that HYP induced cytostructural transformations from polygonal or spindle shaped to flat elongated cells with several split processes reminiscent of neurons and star-like astrocytes in the GBM cell lines (Fig. 2A). These changes suggest that HYP induces morphological differentiation in cultured GBM tumor cells.

**Figure 3:**
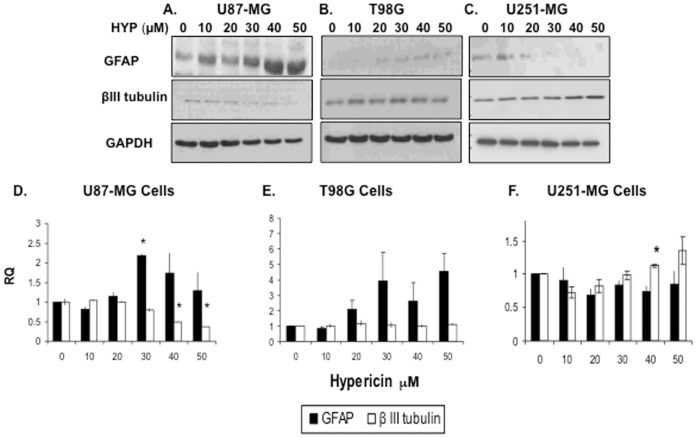
Neuro-glial differentiation antigen expression following cell exposure to HYP, monitored by Western blots. (A) U87-MG cells, (B) T98G cells and (C) U251-MG cells (each well loaded with 20 µg of proteins). (D–F) Effects of HYP on transcriptional expression of GFAP and βIII tubulin genes (mRNA level): (D) in U87-MG cells, (E) in T98G cells and (F) in U251-MG cells. RNA extracted from cells exposed to HYP for 48 hrs was subjected to qRT-PCR analyses. Fold increases calculated relative to RPLPO endogenous control.

Effects of HYP on immunocytochemical neuro-glial differentiation antigen expression in these cells are shown in Fig. 2B (data shown relates to 20 µM HYP dose). In U87-MG cells HYP caused marked increases in glial fibrillary acidic protein (GFAP) and in neuronal nuclei (NeuN) expression, whereas βIII-tubulin levels, a neuron-specific marker, decreased relative to control cells (Fig. 2B). These changes in differentiation marker expression were confirmed in Western blots. βIII-tubulin expression decreased in U87-MG cells in a HYP dose dependent manner whereas GFAP contents were elevated (Fig. 3A). At gene transcription levels HYP upregulated GFAP mRNA expression in a significant manner (P_v_≤0.022) and βIII-tubulin expression was down-regulated (P_v_≤0.044 for the 40 µM HYP group and P_v_≤0.006 for the 50 µM dose) (Fig. 3D). These observations point to induction of glial cell differentiation in U87-MG tumor cells associated with cell proliferation arrest (Fig. 1) in response to HYP in culture.

In T98G cells GFAP, βIII-tubulin and NeuN all increased in response to HYP (Fig. 2C). Western blots confirmed that GFAP cellular levels were elevated in HYP-dose dependent manners (Fig. 3B) and βIII-tubulin levels also showed mild upward changes. Similarly, βIII-tubulin mRNA transcription also showed trends towards increased expression (Fig. 3E) however, the differences were not statistically significant. These patterns suggest that HYP induces neuro-glial differentiation associated with cell proliferation arrest (Fig. 1) in cultured T98G cells.

In U251-MG cells HYP caused elevated expressions of βIII-tubulin and NeuN, whereas GFAP expression declined as evident from the immunofluorescent images (Fig. 2D). Western blots revealed similar HYP-induced decreases in GFAP expression to below detection, whereas βIII-tubulin expression increased (Fig. 3C). Similar trends were noted in the qRT-PCR studies, showing mild βIII-tubulin transcriptional increases (P_v_≤0.028 for the 40 µM HYP group), and trends towards mild decreases in GFAP gene expression (Fig. 3F), (differences not significant). These changes point to induction of neuronal type differentiation with partial down-regulation of cell proliferation in U251-MG cells, showing capability of HYP to overcome the block in U251-MG tumor cell differentiation primarily with respect to differentiation antigen expression.

### Hypericin modulates expression of class I histone deacetylases in glioblastoma cell lines

Similarly to HYP, HDAC inhibitors also induce tumor cell differentiation [17], [27]. We thus, examined if HYP inhibits HDAC activity in GBM cell lines using two approaches: evaluating effects of HYP applied for 30 minutes onto whole cell lysates obtained from untreated cells, and a second approach of measuring total HDAC catalytic activity in lysates from cells pretreated with HYP (as live whole cells) for 72 hrs.

**Figure 4:**
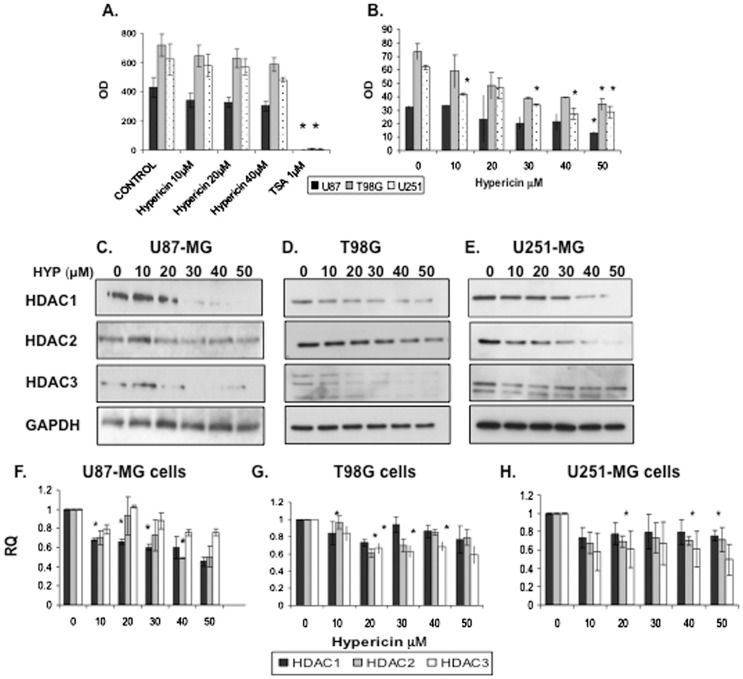
Reduced histone deacetylating activities induced by HYP in GBM cell lines via downregulating cellular expression of class I, histone deacetylases. (A) Effects of HYP applied for 30 minutes onto GBM tumor cell lysates prepared from intact untreated cells. Trichostatin A, 1 µM (TSA) was used as HDAC inhibitor positive control. (B) Total HDAC catalytic activity in lysates obtained from cells pretreated with HYP as intact whole cells (72 hrs). This experimental design quantifies effects of HYP on total HDAC catalytic activity in each cell line. (C–E) Effects of 72 hrs cell exposure to HYP on expression of class I HDACs 1, 2&3 proteins in the cells (Western blots). (C) U87-MG cells, (D) T98G cells, (E) U251-MG cells. GAPDH used for equal loading monitoring, (20 µg of proteins were loaded in each well). (F-H) effects of 48 hrs of cell treatment with HYP on transcription of class I HDAC1, HDAC2 and HDAC3 genes (qRT-PCR analyses). (F) U87-MG cells, (G) T98G cells and (H) U251-MG cells. RPLPO used as endogenous control. Experiments were repeated 3–6 times with a few repeated twice.

HDAC activity in cell lysates administered with 10, 20 and 40 µM HYP (30 min) did not show inhibition of deacetylating catalytic activity in all three cell lines (Fig. 4A), whereas activity was inhibited to ∼99% by 1 µM trichostatin-A used as positive HDACi control (Fig. 4A). HDAC activity in cells pre-exposed to HYP for 72 hrs before lysis declined in T98G cells by 53.2%±13.4% (p≤0.022), in U87-MG cells by 59.6%±10.5% (p≤0.047) and in U251-MG cells by 53.6%±14.9% (p≤0.035) (Fig. 4B). These values, although significant do not reflect HDAC activity inhibition yet suggest that HYP affects cellular HDACs differently.

The hypothesis that in cells pre-exposed to HYP for 72 hrs, cellular HDAC activity decreases due to enzyme deprivation was examined. HDAC1, HDAC2 and HDAC3 protein contents were evaluated in cell extracts using Western blots. Figs. 4C-4E show that pre-exposing cells to HYP caused dose dependent reductions in HDAC1, HDAC2 and HDAC3 cell contents in all three cell lines, although HDAC3 decreases were less consistent in U251-MG and T98G cells (Fig. 4E). The HYP-induced reductions in HDAC protein expression prompted quantification of the HYP effects on transcription of the three *HDAC* genes. qRT-PCR analyses performed on RNA from U87-MG cells exposed to HYP (for 48 hrs) revealed ∼32–55% decreases in *HDAC1* mRNA levels in response to 10–50 µM HYP (significant in the 10–30 µM dose range) (Fig. 4F). Reductions of 10–50% occurred in *HDAC2* (significant with 40 µM HYP). T98G cell *HDAC1* mRNA decreases were mild (5–25%), *HDAC2* decreases ranged between 4–40% (both values significant with 20 µM HYP), and *HDAC3* decreased between 16–41% with 10–50 µM HYP, respectively (significant in 20–40 µM dose range) (Fig. 4G). In U251-MG cells *HDAC1* mRNA levels showed trends toward 22–25% decreases in expression, statistically significant with 50 µM HYP. *HDAC2* mRNA declined 27–32% (significant with 20 and 40 µM HYP). *HDAC3* mRNA levels showed trends towards 40–50% reduced contents (not significant) (Fig. 4H). These studies support our hypothesis that HYP downregulates expression of class I *HDAC*s in GBM cell lines, reducing transcriptions of these genes and resulting in dramatic declines in cellular protein contents with 50–60% loss of cellular deacetylating activities (Fig. 3B).

### Hypericin induces increases in histones H3 & H4 acetylation

Histones H3 and H4 acetylation relaxes chromatin structure, reduces chromatin compaction and increases gene expression, thereby promoting cell differentiation [15], [16]. Histone acetylation is mediated by histone acetyl transferases whereas HDACs reverse this process. Since HYP was found to diminish expression of class I HDACs I, II & III, reduce their activities and induce cell differentiation, we hypothesized that HYP would increase core histone acetylation in the GBM tumor cell lines.

**Figure 5:**
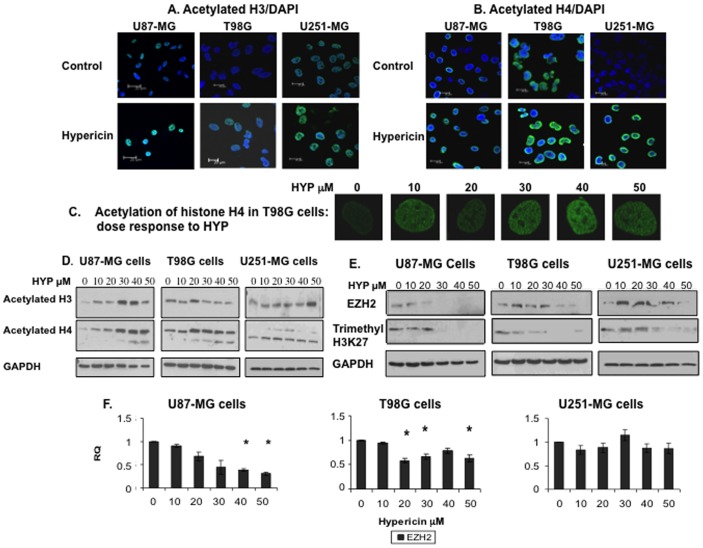
Induction of histones H3 and H4 hyper-acetylation and decreased histone H3-lys27 trimethylation follow GBM cell line treatment with HYP. (A) Histone H3 acetylation levels in nuclei of the GBM tumor cell lines, demonstrated by immunofluorescence following cell treatment with 30 µM HYP and using anti-acetylated histone H3 antibody (1:100 dilution). Upper panel, untreated control cells, lower panel HYP-treated cells. (B) Histone H4 acetylation visualized with antibody to acetylated histone H4 following a similar format. (C) HYP dose-related increases in histone H4 acetylation in T98G cells. (D) HYP effects on histones H3 and H4 protein acetylation determined by Western blots (repeated 3–4 times, 50 µg of proteins loaded into each well). (E) Modulation of EZH2 expression levels in the GBM cell lines by HYP (upper panel. Experiments repeated 2–3 times, 30 µg of proteins loaded into each well). (F) Suppression of EZH2 gene transcription by HYP in the three GBM cell lines (*statistically significant, P≤0.05, student *t-test*).

To determine if HYP affects histones H3 and H4 acetylation, cells exposed to HYP (10–50 µM, 72 hrs) were fixed, stained with fluorescent antibodies directed towards acetylated histones H3 and H4 and visualized by confocal microscopy. Histone H3 acetylation increased following exposure of U87-MG and U251-MG cells to HYP, as determined by immunofluorescence (Fig. 5A, shown for HYP concentration of 30 µM). These findings were validated in Western blots of lysed HYP-treated cells developed with antibodies to acetylated histone H3. Histone H3 acetylation increased in U87-MG cells 5.59 fold following cell exposure to the 30 µM HYP dose and 7.07 fold following exposure to 40 µM HYP as determined by densitometric analyses of the bands in the blots using ImageJ scanning (data shown in Table S3). In U251-MG cells HYP doses of 20 and 30 µM caused 1.6 fold increases in histone H3 acetylation levels (Fig. 5D and Table S3) whereas T98G cells were not affected by HYP in this parameter (Fig. 5A).

Acetylation of histone H4 was found to be markedly increased in all three cell lines as determined by the immunofluorescence assays (Fig. 5B) and confirmed quantitatively in densitometric analyses of the Ac-histone H4 band in the Western blots (Fig. 5D middle panel, quantified in Table S3). U87-MG cells were the most sensitive to HYP and histone H4 acetylation increased with HYP doses as low as 10 µM yielding histone H4 acetylation increases of 3.3 fold, 7.8 fold and 10.2 fold following exposures to 20, 30 and 40 µM HYP, respectively (Table S3). HYP dose-related histone H4 acetylation increases occurred in T98G cells primarily between the 30–50 µM HYP dose levels, Fig. 5C, and in U251-MG cells. Ac-hist-H4 marginal increases of 1.3 fold occurred with the 30 µM HYP dose in T98G cells and 1.9 fold increase for the 30 µM HYP dose in U251-MG cells. Thus, the HYP-dependent histone H4 acetylation increases were observed in Western blots of all three cell lines (Fig. 5D). These findings confirm that the HYP-induced decreases in cellular contents of HDACs are associated with elevated core histone acetylation levels, potentially relaxing chromatin structure and enabling transcription and expression of neuro-glial differentiation related genes.

### Hypericin modulates Polycomb repressive complex-2 catalytic subunit EZH2 expression

The elevated histone acetylation levels induced by exposing GBM cells to HYP prompted us to examine potential effects of HYP on histone methylation patterns. The implicated role for polycomb repressive complex 2 (PRC2) in carcinogenesis, a methyltransferase which tri-methylates histone H3 lysine-27 to H3-K27-3me, led us to focus on this complex. EZH2, the PRC2 catalytic subunit, is abnormally elevated in GBM cells with the highest levels in brain tumors correlating with advanced disease stages and poor prognosis [6]. Because EZH2 forms links with HDACs [7] and DNMTs [8] which modulate its functionality, the abnormal complexes that form in the presence of high EZH2 levels can lead to dysregulated, highly compact chromatin structure that inhibits expression of tumor suppressor and differentiation related genes. It was thus important to examine whether HYP can modulate EZH2 expression levels in the three GBM cell lines and thereby affect histone H3-K27 trimethylation in these cells.

Whole cell lysates were prepared from HYP-treated U87-MG cells (72 hrs) and Western blots developed for EZH2 and H3K27-3me. EZH2 protein expression declined in U87-MG cells to below detection following exposure to ≥30 µM HYP, (Fig. 5E). Similarly, transcription of the EZH2 gene (examined by qRT-PCR) also decreased in a HYP-dose dependent manner, statistically significant with 40–50 µM dose levels (Fig. 5F). H3-K27 trimethylation also decreased in similar manners to below detection following exposure to ≥30 µM HYP, (Fig. 5E).

In T98G cells EZH2 expression was effectively suppressed at the high 40 and 50µM HYP doses (some increases in EZH2 levels also noted with lower HYP doses) (Fig. 5E). The reductions in EZH2 cellular expression led to marked decreases in H3K27-3me levels (Fig. 5E). EZH2 gene transcription (qRT-PCR) was also strongly suppressed by 20-50 µM HYP doses (Fig. 5F).

In U251-MG cells EZH2 contents were only suppressed by the highest HYP dose of 50 µM. EZH2 levels increased following exposure to 10–30 µM HYP (Fig. 5E). EZH2 is another parameter in which U251-MG cell responsiveness to HYP is less robust, however, the resulting H3K27 tri-methylation did decline following exposure to 30–50 µM HYP (Fig. 5E). EZH2 mRNA levels (qRT-PCR) were not significantly affected by HYP (Fig. 5F).

Thus, U87-MG and T98G cells display sensitivities to HYP responding by decreasing EZH2 cellular levels and the consequent histone H3-K27 trimethylation. U251-MG cells are less sensitive to HYP-induced EZH2 downregulation, although histone H3-K27 trimethylation is nevertheless suppressed by HYP.

### Hypericin alters expression profiles of DNA methyltransferases and increases global DNA methylation in GBM cell lines

**Figure 6:**
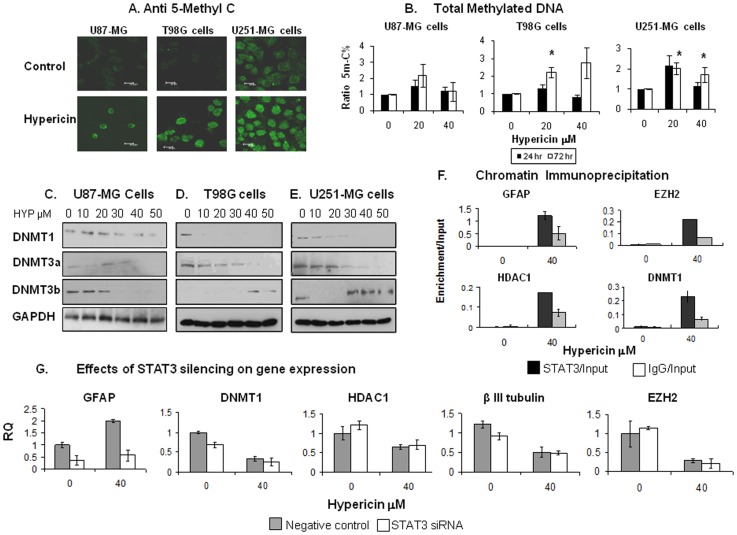
Increases in global DNA methylation following GBM cell exposure to HYP (72 hrs). (A) Monitored as fluorescence of GBM tumor cell nuclei stained with anti 5-methyl cytosine FITC-fluorescent antibody (exposure to 30 µM, HYP). (B) Quantitative analyses (%5-Me-C stained) GBM cell DNA methylation following exposures to HYP. (C-E) HYP effects on DNMT1, DNMT3a and DNMT3b expression in the GBM cell lines examined by Western blots (repeated 3–4 times, two blots repeated twice, 20 µg proteins loaded into each well). (F) Chromatin immunoprecipitation with antibody to STAT3 in GBM cell lines exposed to HYP for 48 hrs. Co-immunoprecipitating DNA was subjected to qPCR evaluations, amplified with primers corresponding to *GFAP, EZH2, HDAC1* and *DNMT1* promoters. Data presented as Enrichment/Input. (G) Effects of *STAT3* silencing using *STAT3*-specific siRNA, on expression of *GFAP, DNMT1, HDAC1, βIII-tubulin* and *EZH2* genes in HYP-treated cells.

Since HYP can readjust aberrant epigenetic properties, down-regulating abnormally high expressions of HDACs and EZH2 in GBM cell lines, we examined if HYP can also affect global DNA methylation in these cells. HYP treated cell lines (72 hrs) were stained with a fluorescently labeled antibody to 5-methyl-cytidine and subjected to immunocytochemical analyses. Marked elevations in global DNA methylation were noted with 30 µM HYP in U251-MG cells and with 40 µM in T98G and U87-MG cells (Fig. 6A). DNA methylation quantification using a methylated DNA quantification kit showed a 2.3 fold increase in global DNA methylation in T98G cells, a 2 fold increase in U251-MG cells following exposure to 20 µM HYP and 2.2 fold trends towards increase in U87-MG cell global DNA methylation with 20 µM HYP, relative to untreated control cells (Fig. 6B).

To understand mechanisms through which HYP can elicit elevations in DNA methylation of GBM cell, the cellular profiles of the DNA methylating enzymes were examined by Western blots. HYP downregulated DNMT1 levels most effectively in T98G cells (Fig. 6D) and caused HYP dose-dependent reductions in U251-MG cell DNMT1 levels (Fig. 6E). DNMT1 was least affected in U87-MG cells (Fig. 6C). HYP dose-dependent reductions were noted with DNMT3a in T98G cells (Fig. 6D) and U251-MG cells (Fig. 6E), yet in U87-MG cells, reduced DNMT3a expression was confined to the highest HYP doses of 40–50 µM (Fig. 6C). DNMT3b expression, however increased in T98G (Fig. 6D) and U251-MG cells (Fig. 6E), though showed a HYP dose dependent reduction in U87-MG cells (Fig. 6C). These studies show that HYP can overcome the global DNA hypomethylation which characterizes GBM tumor cells and modulate DNMT expression profiles in the cells, adding DNA methylation to the epigenetic parameters that are readjusted by this compound.

### Hypericin provokes chromatin conformational changes which culminate in activated STAT3 binding to the promoter and inducing expression of the GFAP gene during U87-MG cell differentiation

Activated signal transducer and activator of transcription-3 (STAT3), is essential for inducing GFAP expression during astrocyte differentiation. STAT3 binds simultaneously to the promoter and to the proximal coding region on exon1 of the *GFAP* gene, promoting neural stem cell differentiation to astrocytes [28], thus playing a central tumor suppressive role [29].

To determine if the HYP-induced astro-glial type differentiation of U87-MG cells and the associated elevated *GFAP* expression follow normal pathways, we examined if HYP provokes STAT3 activation, nuclear translocation and binding to consensus sequences on *GFAP, HDAC1, EZH2* and *DNMT1* promoters. Chromatin immunopercipitation (ChIP) analyses were used in these studies. Sheared chromatin from HYP-treated cells, immunoprecipitated with antibody to STAT3 was subjected to quantitative ChIP analyses. The qRT-PCR assays which were performed using primers to these four genes show that exposure to 40 µM HYP resulted in enrichment of STAT3 binding to promoters of *HDAC1, EZH2, DNMT1* and *GFAP* genes (Fig. 6F). However, since HYP treatment of U87-MG cells downregulates HDAC1, EZH2 and DNMT1 expression, whereas expression of GFAP is markedly elevated we examined the role of STAT3 in these HYP induced modulations by silencing *STAT3* expression using *STAT3*-specific siRNA. qRT-PCR analyses performed with primers to the above mentioned genes and to *βIII-tubulin*, a neuronal differentiation gene not induced in U87-MG cells and used as a control, show that *STAT3* silencing did not affect *HDAC1, EZH2, DNMT1* or *βIII-tubulin* gene expression with the sole effects being the responses to HYP (Fig. 6G). With *GFAP* however, *STAT3* silencing abrogated the HYP-induced elevation in GFAP expression (Fig. 6G, left exhibit). These observations suggest that the HYP-induced differentiation of U87-MG cells follows the expected normal embryonal path of *GFAP* induced expression which involves the STAT3 pathway without regulating expression of neither *HDAC1* nor *EZH2* or *DNMT1* genes.

## Discussion

A promising potential candidate for modifying multiple aberrant epigenetic equilibriums and achieving therapy for GBM is HYP. This redox reactive compound [30] with redox potentials slightly lower than most cellular electron transfer mediators [31] displays multiple biological activities. The most important HYP activity is induction of Hsp90 polyubiquitination [18], [19] which accelerates degradation of this chaperone and the plethora of Hsp90 client proteins in tumor cells.

Hsp90 has been implicated in forming links between biochemical chaperone activities and epigenetic gene regulation in developing organisms [22]. This chaperone interacts with components of chromatin remodeling complexes in yeast [21], and SMYD3, the human histone H3 lysine-4 methyltransferase, requires Hsp90 for its activity [32]. Hsp90 also binds and stabilizes DNMT1, preventing DNMT1 ubiquitination [33].

By disrupting Hsp90, HYP can touch upon the link between Hsp90 chaperoning and epigenetic gene regulating activities. We show here that HYP targets several different chromatin-modifying activities. These include induction of marked global hyperacetylations of histones H3 and H4 (Fig. 5A-E), which can otherwise be induced by HDACi. HYP however, is not an HDACi (Fig. 4A) and acts by downregulating class I HDACs 1, 2 and 3 expression, (Fig. 4A-E). The resulting cellular deprivation of HDACs may promote the observed elevated histone acetylation levels thereby relaxing the characteristically high tumor cell chromatin compaction [5]. HYP can also interfere with recruitment of HDACs to silencing complexes on promoters, thereby facilitating induction of transcriptional gene activation. By down-regulating EZH2 in U87-MG and T98G cells, this action of HYP reduces histone H3-K27 tri-methylation and further relaxes chromatin structure. Access is thus rendered to transcription-promoting complexes to reach promoters of tumor suppressors, cell replication checkpoints and differentiation associated genes. HYP modulates their expression profiles and GBM tumor cell cyto-architecture is modified to astrocyte-like and neuron-like morphologies associated with modified neuro-glial differentiation antigen expression and exit from cell proliferation cycles. Thus in this proof of concept paper we show that HYP can induce post-mitotic tumor cell differentiation in GBM cell lines, mimicking the differentiation caused by HDACi [17], [27], [34], although via a different and more complex mechanism.

HYP mediates the various epigenetic gene expression regulating effects described here without entering the cell nucleus at any time. HYP concentrates in the GBM cell cytoplasm, primarily in the perinuclear golgi apparatus (not shown) where it binds to hsp90 [18].

The HYP-induced cell differentiation patterns vary among the three analyzed GBM cell lines. U251-MG cells undergo neuronal differentiation, U87-MG cells astroglial and T98G cells combined neuro-glial differentiation. Glial marker GFAP expression, elevated in HYP-treated U87-MG cells appears to follow the normal transcriptional activating mechanism mediated by STAT3. However, a more significant disadvantage emerges from the cell replication analyses. It involves the linkage between cell differentiation antigen expression and exit from cell proliferation, the most important aspect of tumor cell differentiation relevant to anti-cancer therapy. Although, normal differentiation is tightly linked to cessation of cell proliferation in most tissues, this is apparently not granted in tumor cell differentiation. Chromatin landscape changes controlling expression of differentiation-related features are often linked to proliferation-controlling checkpoint genes, generating complete post-mitotic tumor cell differentiation as that induced by HYP in U87-MG and T98G cells (Fig. 1). However, replication shutdown seems less robust in HYP-induced U251-MG cell differentiation (Fig. 1), albeit strongly elevated expression of differentiation antigens (Fig. 2). These findings point to potential therapeutic flaws in incorporating epigenetic modulators such as HYP to tumor cell differentiation therapy in GBM.

We were able to firm U251-MG cell proliferation arrest by combining HYP with HDACi as Trichostatin A or valproic acid, potentially diminishing this problem, however this has yet to be confirmed in animal models *in-vivo* (outside the scope of this paper).

Variability in U251-MG cell responses to HYP compared to U87-MG and T98G cell responses may also involve the effects of this compound on EZH2. HYP downregulates EZH2 in U87-MG and T98G cells (at the high doses which were studied) reducing EZH2-mediated histone H3-K27-3me levels (Fig. 5E). In U251-MG cells exposed to HYP EZH2 becomes elevated but nevertheless H3K27 tri-methylation declines. This may be explained by the PRC2 physical association with HDAC1 and HDAC2 [7] and HDACi as TSA interfere with the PRC2-mediated gene silencing process [7]. Since HDAC activities are instrumental in maintaining transcriptional silencing, the reductions in cellular contents of HDACs caused by HYP can interfere with PRC2 complex activities even when EZH2 is not effectively downregulated as in U251-MG cells. Variations in acetylation of other histone lysines (as H3-K9, H3-K14 or H4-K8) and modulating equilibriums of other histone-lys methyltransferases – demethylases [35] can also unequally adjust local histone codes for silencing, affecting various genes differently.

DNMTs participate with HDACs and EZH2 in highly regulated functional complexes [36]. In GBM cells these interactions appear to be abnormal due to high expressions of HDACs and EZH2. HYP may correct some of these aberrations possibly by down-regulating HDACs and EZH2. HYP also alters the aberrant expression profiles of DNMTs described for GBM cells as up-regulation of DNMT1 and down-regulation of the de novo DNMT3a [12]. We show that in U87-MG, T98G and U251-MG cells HYP down-regulates DNMT1 expression and in the latter two lines up-regulates DNMT3b (Fig. 6C-E). The DNMT profiles that are generated are incompatible with the abnormal GBM cell profiles of these enzyme and in U87-MG cells DNMT3b expression is down-regulated in a HYP dose dependent manner. The HYP-modified DNMT expression profiles are likely to elicit functional DNMT activity changes which may bear closer similarities to those of normal cells. Indeed HYP-induces increases in global DNA methylation, which may help stabilize the GBM cell genome.

The induction of GBM tumor cell differentiation by HYP has yet to be verified in animal models *in vivo*. Such confirmation will bear clinical implications as HYP was indeed reported to show efficacy against GBM in a Phase I/II clinical trial when evaluated in recurrent, progressive GBM patients in 6 North American Centers. Despite this most difficult to treat advanced GBM stage, 21.4% of patients were treatment responders and 40% completed the 3 month trial with median survival of 26 weeks [37]. Twelve patients continued on compassionate therapy beyond the trial and eight survived ≥6–34 months (mean survival 20.8 months) [37]. Suppression of disease progression occurred without tumor disappearance primarily achieving stable disease and in 2 patients also partial responses (≥50% reduction in tumor volume) [37]. This outcome suggests that HYP effectively modified the biological properties of GBM cells. We believe that these changes reflect induction of post-mitotic tumor cell differentiation similarly to the HYP effects on cultured GBM cell lines. HYP has also been reported by other groups to induce tumor cell differentiation in human HL60 leukemia [38] and U-937 histiocytic lymphoma [39] cells.

Furthermore, reductions in class I HDAC expression may also play important roles in the anti-GBM activities of HYP as strong expressions of HDACs 1&2 have been key findings in 283 GBM tumor biopsy samples and noted to increase during tumor recurrence and progression [40]. HYP may also diminish risks associated with abnormally elevated EZH2 levels observed in several tumors including GBM and suggested to correlate with advanced disease stage and poor prognosis [6].

## Supporting Information

Table S1 **Sequences of primers used to amplify the various genes that were analyzed in the Real Time Quantitative RT-PCR Assays.**
(DOC)

Table S2 **Sequences of primers used in the Chromatin Immunoprecipitation (ChIP) Analyses.**
(DOC)

Table S3 **Densitometric analyses of data shown in Western blots in** **Figures 4** **–** **6** **depictsing the effects of cell exposure to hypericin on:** (1). Expression of class I HDACs 1, 2&3 proteins in: U87-MG cells, T98G cells an U251-MG cells. (2). Histones H3 and H4 protein acetylation determined by Western blots. (3). Quantification of DNMT1, DNMT3a and DNMT3b expression in the GBM cell lines by Western blots. Numbers are given relative to the GAPDH housekeeping gene), using ImageJ.
(DOC)
