# Supplementary material for: Unique Anti-Glioblastoma Activities of Hypericin Are at the Crossroad of Biochemical and Epigenetic Events and Culminate in Tumor Cell Differentiation
Source: PLoS One. 2013 Sep 16;8(9):e73625. doi: 10.1371/journal.pone.0073625 (PMC3774735; doi:10.1371/journal.pone.0073625)
Supplement: Table S1 — Sequences of primers used to amplify the various genes that were analyzed in the Real Time Quantitative RT-PCR Assays. (DOC) [file pone.0073625.s001.doc]

**Table S1: Primers used in the Real Time Quantitative RT-PCR A**ssays

| Primers | Gene Name |
| --- | --- |
| TGTTAACTACCCGCTCCGAGA  TGGACATGACCGGCTTGAA | HDAC1(F)  HDAC1(R) |
| GGCACAGGAGACTTGAGGGATAT  CAGCATAGTATT TGCCTT TTCCAG | HDAC2(F)  HDAC2(R) |
| GAGTGGCCGCTACTACTGTCTGA  AAAGGTGCTTGTAACTCTGGTCATC | HDAC3(F)  HDAC3(R) |
| AGAACGCCTTTAAGCGCCG  CCGTCCACTGCCACCAAAT | DNMT1(F)  DNMT1(R) |
| AGGAGTATTTTGCGTGTGTA  TCTTGGTGTTTTATTATGTTTTGTGTT | DNMT3A(F)  DNMT3A(R) |
| TTACCACCTGCTGAATTACTCAC  CATCAATCATCACTGGATTACACTC | DNMT3B(F)  DNMT3B(R) |
| CACGCAGTATGAGGCAATGG  TCTGCAAACTTGGAGCGGTAC | GFAP(F)  GFAP(R) |
| GGCCAAGTTCTGGGAAGTCA  TCCGAGTCGCCCACGTAG | β III tubulin(F)  β III tubulin(R) |
| GCCAGACTGGGAAGA AAT  TGTGTTGGAAAATCCAAGT | EZH2(F)  EZH2(R) |
| GAGGGTGTCCGCAATGTTG  TCAGCAAGTGGGAAGGTGTA | RPLPO(F)  RPLPO(R) |
