# Supplementary material for: Unique Anti-Glioblastoma Activities of Hypericin Are at the Crossroad of Biochemical and Epigenetic Events and Culminate in Tumor Cell Differentiation
Source: PLoS One. 2013 Sep 16;8(9):e73625. doi: 10.1371/journal.pone.0073625 (PMC3774735; doi:10.1371/journal.pone.0073625)
Supplement: Table S2 — Sequences of primers used in the Chromatin Immunoprecipitation (ChIP) Analyses. (DOC) [file pone.0073625.s002.doc]

**Table S2: Primers used in the Chromatin Immunoprecipitation (ChIP) A**nalyses

| Primers | Gene Name |
| --- | --- |
| TCTCCGTAAAATCAGAAAGTTGG  AGGAAAGCAGCTCTGTAAACT | HDAC1 (F)  HDAC1 (R) |
| ATTCTGTGTATACTCAAGATTTTCTAG  CAATCCAACCTCCATCTATTTGC | DNMT1 (F)  DNMT1 (R) |
| GGGACGGGACAGACACAA  CTCCGGAGACACAGTCCG | EZH2 (F)  EZH2 (R) |
| GCTTCCTGGAACAGCAAAAC  AGGTCCTGTGCCAGATTGTC | GFAP (F)  GFAP (R) |
