# Supplementary material for: Unique Anti-Glioblastoma Activities of Hypericin Are at the Crossroad of Biochemical and Epigenetic Events and Culminate in Tumor Cell Differentiation
Source: PLoS One. 2013 Sep 16;8(9):e73625. doi: 10.1371/journal.pone.0073625 (PMC3774735; doi:10.1371/journal.pone.0073625)
Supplement: Table S3 — Densitometric analyses of data shown in Western blots in Figures 4 – 6 depictsing the effects of cell exposure to hypericin on: (1). Expression of class I HDACs 1, 2&3 proteins in: U87-MG cells, T98G cells an U251-MG cells. (2). Histones H3 and H4 protein acetylation determined by Western blots. (3). Quantification of DNMT1, DNMT3a and DNMT3b expression in the GBM cell lines by Western blots. Numbers are given relative to the GAPDH housekeeping gene), using ImageJ. (DOC) [file pone.0073625.s003.doc]

**Table S3 – Densitometric analyses of data shown in Western blots in Figures 4-6 (relative to GAPDH), using ImageJ**

**U87-MG cells**

| **Gene** | **Hypericin µM** | **0** | **10** | **20** | **30** | **40** | **50** |
| --- | --- | --- | --- | --- | --- | --- | --- |
| **HDAC1 (Fig. 4C)** | | 0.6589 | 0.6767 | 0.3675 | 0.0926 | 0.0851 | 0.0422 |
| **HDAC2 (Fig. 4C)** | | 0.4775 | 0.4881 | 0.2153 | 0.2019 | 0.2703 | 0.2897 |
| **HDAC3 (Fig. 4C)** | | 0.2578 | 0.6607 | 0.1002 | 0.0372 | 0.1106 | 0.1121 |
| **DNMT1 (Fig. 6C)** | | 0.7738 | 0.9430 | 0.6202 | 0.5171 | 0.4635 | 0.4398 |
| **DNMT3a (Fig. 6C)** | | 0.5417 | 0.3785 | 0.8396 | 0.7240 | 0.4618 | 0.4169 |
| **DNMT3b (Fig. 6C)** | | 0.8471 | 0.8523 | 0.6246 | 0.6337 | 0.4472 | 0.5039 |
| **Ac-Histone H3 (Fig. 5D)** | | 0.2382 | 0.4954 | 0.4510 | 1.3327 | 1.6857 | 0.8067 |
| **Ac-Histone H4 (Fig. 5D)** | | 0.1618 | 0.3730 | 0.5376 | 1.2550 | 1.6486 | 1.6931 |

**T98G cells**

| **Gene** | **Hypericin µM** | **0** | **10** | **20** | **30** | **40** | **50** |
| --- | --- | --- | --- | --- | --- | --- | --- |
| **HDAC1 (Fig. 4D)** | | 0.7173 | 0.3728 | 0.3355 | 0.2949 | 0.2643 | 0.3793 |
| **HDAC2 (Fig. 4D)** | | 0.658 | 0.6125 | 0.5497 | 0.4635 | 0.3503 | 0.3349 |
| **HDAC3** **(Fig. 4D)** | | 0.908 | 0.9419 | 0.7482 | 0.7479 | 0.8663 | 1.0245 |
| **DNMT1 (Fig. 6D)** | | 0.5800 | 0.4510 | 0.3407 | 0.4010 | 0.3330 | 0.3710 |
| **DNMT3a (Fig. 6D)** | | 0.7418 | 0.4677 | 0.3076 | 0.2213 | 0.0811 | 0.0686 |
| **DNMT3b (Fig. 6D)** | | 0.3972 | 0.3411 | 0.4175 | 0.3928 | 0.6785 | 0.4909 |
| **Ac-Histone H3 (Fig. 5D)** | | 0.9496 | 0.6918 | 0.9156 | 0.5165 | 0.5614 | 0.4654 |
| **Ac-Histone H4 (Fig. 5D)** | | 0.5973 | 0.6515 | 0.9946 | 0.8108 | 0.8396 | 0.5582 |

**U251-MG cells**

| **Gene** | **Hypericin µM** | **0** | **10** | **20** | **30** | **40** | **50** |
| --- | --- | --- | --- | --- | --- | --- | --- |
| **HDAC1 (Fig. 4E)** | | 0.6911 | 0.5502 | 0.5501 | 0.4057 | 0.2077 | 0.1668 |
| **HDAC2 (Fig. 4E)** | | 0.2963 | 0.1796 | 0.1679 | 0.1073 | 0.0466 | 0.0427 |
| **HDAC3 (Fig. 4E)** | | 0.7817 | 0.5612 | 0.5495 | 0.6579 | 0.5642 | 0.9397 |
| **DNMT1 (Fig. 6E)** | | 0.3631 | 0.2542 | 0.1112 | 0.1587 | 0.1818 | 0.2054 |
| **DNMT3a (Fig. 6E)** | | 1.2094 | 0.6248 | 0.9223 | 0.2898 | 0.1901 | 0.2018 |
| **DNMT3b (Fig. 6E)** | | 0.3630 | 0.2160 | 0.1580 | 0.8061 | 0.8709 | 0.8704 |
| **Ac-Histone H3 (Fig. 5D)** | | 0.2191 | 0.2253 | 0.3419 | 0.3525 | 0.2405 | 0.6183 |
| **Ac-Histone H4 (Fig. 5D)** | | 0.5125 | 0.8598 | 1.2549 | 0.9907 | 0.8075 | 0.7862 |
